# Annual acknowledgement of reviewers

**DOI:** 10.1186/s12867-016-0058-8

**Published:** 2016-02-19

**Authors:** Timothy R. Sands

**Affiliations:** BioMed Central, Floor 6, 236 Gray’s Inn Road, London, WC1X 8HB UK

## Abstract

The editors of *BMC Molecular Biology* would like to thank all our reviewers who have contributed to the journal in Volume 16 (2015).

## Contributing reviewers

**Simak Ali**

USA

**Antonio Alonso**

Spain

**Sam Alsford**

USA

**Mario Arteaga-Vázquez**

Japan

**Federico Avila-Moreno**

Mexico

**Agnieszka Bialkowska**

Japan

**Antony Braithwaite**

New Zealand

**Adam Brown**

USA

**David Carter**

UK

**Thomas Caspari**

UK

**Che-Chang Chang**

USA

**Young Lae Cho**

South Korea

**Katrin Chua**

USA

**Fabio Ciccarone**

USA

**Anita Corbett**

USA

**Alejandra Covarrubias**

USA

**Agnes Csiszar**

Austria

**Paul Delgado-Olguín**

Australia

**William Dunphy**

USA

**Fiona Furlong**

UK

**Niels Gehring**

Australia

**Thomas George**

Malaysia

**Homa Ghalei**

USA

**Thomas Greither**

USA

**Channabasavaiah Gurumurthy**

USA

**Michelle Hastings**

Taiwan

**Gyorgy Hutvagner**

Australia

**Bulent Icgen**

Turkey

**Junji Itou**

Japan

**Rory Johnson**

Spain

**Inácio de L. M. Junqueira de Azevedo**

Spain

**Brent Kaiser**

Italy

**Eiji Kakizaki**

Japan

**Martin Kluger**

USA

**Wilfried Kues**

Austria

**Takahiro**  **Kunisdata**

Taiwan

**Frederik Leliaert**

USA

**Alfonso León-Del-Río**

China

**Lisa Lincz**

Italy

**James Lister**

Germany

**Laura Lossi**

Italy

**David Meek**

USA

**Lourdes Mengual**

Spain

**Marija Mihailovich**

Italy

**Norbert Nass**

Thailand

**Paolo Neviani**

USA

**Michael O’Connor**

USA

**Anand Pai**

USA

**Michael Paulaitis**

Mexico

**Nicholas Redshaw**

UK

**Deborah Robertson**

USA

**Remo Rohs**

USA

**Ponlapat Rojnuckarin**

New Zealand

**Andrei Seluanov**

USA

**Gregory Shipley**

USA

**Lea Starita**

USA

**Padraig Strappe**

Germany

**Per Sunnerhagen**

Sweden

**David Svec**

Czech Republic

**Kaixiong Tao**

China

**Toshiki Tsurimoto**

Japan

**Viviana Valadez**

Mexico

**Juan Valcarcel**

Germany

**Lionel Verdoucq**

USA

**Pablo Vivas**

Puerto Rico

**Sunil Wadhwa**

USA

**Ju-Ming Wang**

Malaysia

**Chunyao Wei**

USA

**Ulrich Friedrich Wellner**

Brazil

**Jacek Wower**

UK

**Jianchang Yang**

USA

**Fung Shin Yee**

UK

**Je-Hyun Yoon**

Australia

**Xiao-Ning Zhang**

Sweden

**Man Zhou**

Mexico

**Victor Zhurkin**

USA

